# Sexual function and postpartum depression 6 months after attempted operative vaginal delivery according to fetal head station: A prospective population-based cohort study

**DOI:** 10.1371/journal.pone.0178915

**Published:** 2017-06-07

**Authors:** Guillaume Ducarme, Jean-François Hamel, Stéphanie Brun, Hugo Madar, Benjamin Merlot, Loïc Sentilhes

**Affiliations:** 1 Department of Obstetrics and Gynecology, Centre Hospitalier Departemental, La Roche sur Yon, France; 2 Clinical Research Center, Angers University Hospital, Angers, France; 3 Department of Obstetrics and Gynecology, Bordeaux University Hospital, Bordeaux, France; Royal Children's Hospital, AUSTRALIA

## Abstract

**Objective:**

To evaluate the effect of the fetal head station at attempted operative vaginal delivery (aOVD), and specifically midpelvic or low aOVD, on female and male sexual function and symptoms of postpartum depression (PPD) at 6 months.

**Design:**

Prospective population-based cohort study.

**Setting:**

1,941 women with singleton term fetuses in vertex presentation with midpelvic or low aOVD between 2008 and 2013 in a tertiary care university hospital.

**Methods:**

Symptoms of female sexual dysfunction using the Pelvic Organ Prolapse/Urinary Incontinence/Sexual Function Short Form Questionnaire (PISQ-12), symptoms of PPD using the Edinburgh Postnatal Depression Scale (EPDS) score, symptoms of male sexual dysfunction using the International Index of Erectile Function (IIEF-15) and perineal pain were assessed 6 months after aOVD. We measured the association between midpelvic or low aOVD and symptoms of female and male sexual function and symptoms of PPD at 6 months using multiple regression and adjusting for demographics, and risk factors of sexual dysfunction, symptoms of PPD and perineal pain with adjusted odds ratios (aORs) and 95% confidence intervals (95% CI).

**Results:**

The study included 907 women (46.7%) who responded to the questionnaire; 18.4% (167/907) had midpelvic aOVD, and 81.6% (740/907) low. Most women (873/907 [96.3%]) of those with partners reported sexual activity at 6 months. No significant difference was observed for PISQ-12, EPDS, IIEF-15 scores and perineal pain between mid and low pelvic groups. Compared with low pelvic aOVD, midpelvic aOVD was not significantly associated with either female or male sexual dysfunction (p = 0.89 and p = 0.76, respectively), or maternal symptoms of PPD (p = 0.83). Perineal pain significantly increased the risk of male and female sexual dysfunction and maternal symptoms of PPD at 6 months (p = 0.02, p = 0.006, and p = 0.02, respectively).

**Conclusion:**

Midpelvic compared with low pelvic aOVD was not associated with an increase in sexual dysfunction, nor with symptoms of PPD at 6 months.

## Introduction

No obstetrician wants to deliver a fetus already or still high in the birth canal; this situation is risky for mother and child. But in some situations (long labor, suspicion of fetal distress), obstetricians are face with a choice and can feel required, because of the obstetric situation, to attempt a potentially difficult operative vaginal delivery (OVD) or perform a cesarean at full dilatation, each with immediate and long-term inherent maternal and neonatal risks. Furthermore, when the fetus is at low pelvic station, OVD is not discussed for maternal or fetal indication, and must be preferred to cesarean delivery [1]. We previously reported that midpelvic attempted operative vaginal delivery (aOVD) was not associated with a higher rate of severe short-term maternal and neonatal morbidity than attempted low pelvic delivery supporting the continued use of midpelvic delivery in appropriately selected candidates [2]. Nonetheless, midpelvic OVD may subsequently have hidden mid- and long-term effect, that may justify to privilege a cesarean delivery rather than an OVD when the fetus is at midpelvic. Thus, it is crucial to assess mid and long-term maternal outcome after midpelvic aOVD. Our previous report that neither urinary nor anal incontinence differed at 6 months among women who had midpelvic and low pelvic aOVD suggests that midpelvic aOVD is an appropriate option [3].

Postpartum depression (PPD) is determined by several determinants, including consequences of obstetrical factors, such as mode of delivery, pain during delivery [4,5], parity, pregnancy or delivery complications [6–8]. Some studies suggest that OVD, compared to spontaneous vaginal delivery, are associated with a higher risk for symptoms of PPD [9,10], some do not [11,12]. Discrepancies may be explained by methodological differences.

Quality of female sexual function in postpartum period is dependent of several different factors, including cultural, sexological, organic, neurological patterns, psychological wellbeing [13], and perinatal events, including perineal pain, episiotomy, symptoms of PPD [14–16]. OVD has not clearly been reported as an independent risk factor for alteration of sexual function [13,15,17,18]. Recently, Barbara et al. [19] reported that OVD (n = 45) might be associated with poorer female sexual functioning, and specifically poorer scores on arousal, lubrication, orgasm, and global sexual functioning compared with a cesarean section group (n = 92) and lower orgasm scores compared with a spontaneous vaginal delivery group (n = 132) [19]. But, no conclusions can be drawn from this study regarding the high rate of episiotomies, the impact of pelvic floor trauma (perineal laceration or episiotomy) on sexual functioning, the small sample size and the absence of multivariate models controlled for confounders. Concerning male sexual function, a review of 59 studies noted that data about fathers, sexual activities and feelings in postpartum period are scarce [20]. Nearly a third of men reported lower sexual desire along with decreases in sexual behavior during postpartum period [21]. The association between the mode of delivery and sexual function have been already analyzed [13,15,17,18] but, to our knowledge, none of these studies has specifically studied female and male sexual function and depressive symptoms in postpartum period according to the fetal head station at aOVD.

We aimed to analyse female sexual function, maternal symptoms of PPD and male sexual function at 6 months according to the fetal head station at aOVD (midpelvic compared to low pelvic aOVD), and to analyse the risk factors of sexual dysfunctions and symptoms of PPD on a large prospective population-based cohort study of women who underwent an OVD, using multivariate models adjusting for potential confounders.

## Material and methods

### Study sample

All participants were told about the study and were given oral information during pregnancy. Verbal consent was obtained by the medical team in charge of the study before inclusion, women and men were consented to participation after delivery in the labor ward or in the maternity unit. Written consent was not required for prospective population-based cohort study according to the French law, and the study protocol and this consent procedure were approved by the Institutional Review Board at the Angers University Hospital, France, on November 2008 (Study ID: 2008), before the beginning of the study [2].

Our post hoc analysis were done using data from a prospective population-based cohort study including all women with an aOVD in a tertiary care university hospital from December 2008 through October 2013. Inclusion criteria was defined by the placement of at least one blade for forceps or spatula or a vacuum, regardless of its success (i.e., whether delivery was finally vaginal or cesarean), and a live singleton pregnancy in vertex presentation at term (> 37 weeks of gestation) [2]. Exclusion criteria were multiple gestations, fetal growth restriction (FGR), defined as <10^th^ percentile for gestational age on Hadlock curves [22,23], a known congenital anomaly, vaginal breech delivery, and the absence of fetal station information according to the American College of Obstetricians and Gynecologists (ACOG) classification [24]. Specifically, station was defined by the level of the leading bony point of the fetal head in centimetres at or below the level of maternal ischial spines (0 and +1 = midpelvic; +2 and +3 = low; +4 and +5 = outlet) [24]. As described in detail previously [2], the hospital had 19,786 deliveries during the study period: 15,836 (80.0%) were vaginal, including 2,153 (13.6%) successful OVD, and 3,950 (20.0%) were cesarean deliveries, including 39 (0.2% of all deliveries and 1% of all cesarean deliveries) after failed OVD. There were thus 2,192 deliveries with an aOVD: successes 98.2% and failures 1.8%. However, 28 neonates were twins (n = 14 women), 26 were preterm, and 14 were small for gestational age and were therefore excluded. Therefore, our sample comprised 2,138 deliveries with an aOVD: 18.3% (n = 391) midpelvic, 72.5% (n = 1,550) low, and 9.2% (n = 197) outlet. Among all women with a fetus at midpelvic station at delivery, only 17 (4.2%) had a cesarean delivery without an operative vaginal attempt [2].

As described in detail previously [2], pre-specified study design was to analyse severe short-term maternal and neonatal morbidity after aOVD according to the fetal head station using the ACOG classification, specifically to compare severe short-term maternal and neonatal morbidity associated with midpelvic and low pelvic aOVDs, and to prospectively analyse mid- and long-term maternal complication (pelvic floors disorders, sexual dysfunction, maternal postpartum depressive symptoms at 6 months) and children development at 5 years, specifically associated with midpelvic and low pelvic aOVDs. Two cohorts of women were assessed separately 6 months after aOVD: those with a midpelvic aOVD (n = 391; 20.6%), and a lowpelvic aOVD (n = 1,550; 79.4%).

### Measures

Informations about sexual function in men and women, and maternal symptoms of PPD were obtained from a questionnaire sent 6 months after delivery. A second mailing was sent to the women from whom we received no response. Female sexual function was assessed with the Pelvic Organ Prolapse Urinary Incontinence Sexual Questionnaire (PISQ-12) [25–27]. The PISQ-12 is a self-administered questionnaire with responses measured on a 5-point Likert scale ranging from 0 (always) to 4 (never), which have been already used to evaluate female sexual function in postpartum period [26], and seemed to be appropriate in a population suffering from postpartum pelvic floor disorders [3]. The questionnaire contains 12 items divided into three domains: behavioral-emotive (items 1–4), physical (items 5–9) and partner-related (items 10–12). The behavioral/emotive domain evaluates sexual desire, frequency of sexual activity, and orgasmic capabilities, the physical domain assesses more directly the effect of urinary incontinence or prolapse on sexual function, and the partner-related domain assesses the patient’s perception of her partner’s response to the effect of her pelvic floor disorder on their sexual functioning [25–27]. Items 1–4 are reversely scored and a total of 48 is the maximum score; higher scores indicate better female sexual function. The PISQ-12 is reported as a single sexual function score. It does not report the separate domains. This questionnaire has been validated in French to evaluate female sexual function [28]. Participants were considered sexually active if they reported sexual activity with a partner in the prior month.

As previously reported [3,29], perineal pain was evaluated through the question: “Do you have chronic perineal pain (perineum designates the skin and muscle around the vaginal and anal outlets)?”, dyspareunia through the question: “Do you experience pain during sexual intercourse?”. These two questions were dichotomous with two possible answers: “yes” and “no”, with an option for women who do not have intercourse in the prior month [3,29]. Episiotomy complications were evaluated through the question: "Do you have any complications concerning your episiotomy (hematoma, abscess, scar disunion, surgery)?", and was defined by the existence of at least one of the following criteria: hematoma, abscess, scar disunion, or required surgery for episiotomy [3,29].

Maternal symptoms of PPD was assessed using the French version of the Edinburgh Postnatal Depression Scale (EPDS) [30,31]. The EPDS is a 10-item self-report scale, it sums score ranges from 0 to 30 points, with higher score indicating more symptoms. EPDS has good sensitivity and specificity for identifying probable clinical postpartum depression in community samples [32,33]. In accordance with recent studies, a score of ≥12 on the EPDS was used as a measure of symptoms of PPD [12,34].

The effect of the delivery on quality of male sexual relations was assessed by using the International Index of Erectile Function (IIEF-15) [35], which has been already used to evaluate male sexual function in post-partum period [36]. The partner of the included women answered to the IIEF-15 questionnaire, which is a a self-administered questionnaire with responses measured on a 5-point Likert scale. The questionnaire addresses the relevant domains of male sexual function and contains 15 items divided into five domains: erectile function (Q1-Q5, Q15), orgasmic function (Q9-10), sexual desire (Q11,12), intercourse satisfaction (Q6-Q8), and overall satisfaction(Q13-14) [35]. Higher scores indicate better male sexual function [34]. The French linguistic version of the IIEF-15 was used [37]. The questionnaire used for the study is available (S1 Questionnaire).

The principal endpoints were PISQ-12 for female sexual function and EPDS score≥12 for maternal symptoms of PPD. The secondary endpoint was IIEF-15 for male sexual function. For this study, we hypothesized that women with midpelvic aOVD would not have poorer sexual function and more symptoms of PPD compared to low pelvic aOVD, and male sexual function was not altered after midpelvic aOVD compared to low pelvic aOVD.

### Statistical analysis

Continuous data were described by their means ± standard deviations and compared by t-tests (or Mann-Whitney tests when appropriate), and categorical data were described by percentages and compared by chi-square tests (or Fisher exact tests when appropriate). Univariate and multivariate logistic regression were used for studying the association between maternal symptoms of PPD at 6 months and aOVD classification. Univariate and multivariable linear regression analysis were used to study the association between female and male sexual function at 6 months (considered as a continuous variable) and aOVD classification. Multivariate models were build using a stepwise procedure based on the Akaike criterion [38,39], the covariate "fetal head station" being systematically forced into the considered models. Confounders associated in univariate analysis at a 0.2 level were included in this stepwise procedure. To check the fit of the multivariate models, we studied the studentized residuals. Standardized measures of Cohen's d effect sizes were analysed in all comparisons (female sexual function, maternal symptoms of PPD, male sexual function), and specifically, midpelvic compared to low pelvic aOVD. The association between the different scores (PISQ, IIEF and EPDS) were also studied through the Pearson’s correlation coefficient. STATA 13.1 software (StataCorp, College Station, TX) was used for all analyses. Statistical significance was defined as a *P* value < 0.05.

## Results

**Fig 1:**
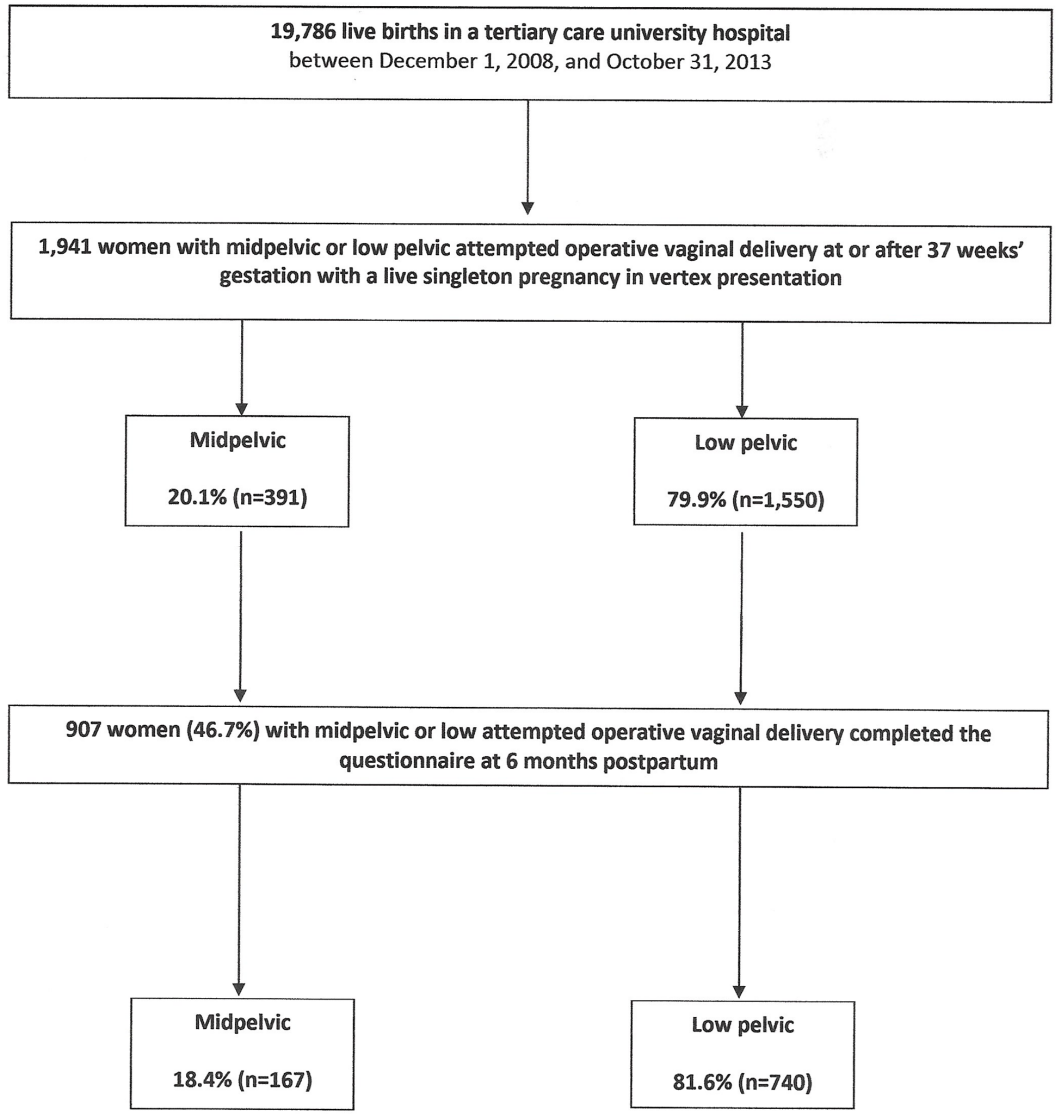
Cohort flowchart.

Six months after delivery, 907 women (46.7%) of the 1,941 deliveries with an aOVD completed the questionnaire: 18.4% (n = 167) had been midpelvic, and 81.6% (n = 740) low attempted deliveries (Fig 1). The difference between respondents and non-respondent were statistically significant in terms of maternal age at delivery, marital status, severe neonatal morbidity, rates of NICU transfer and prolonged hospitalization in NICU (p<0.05) (S1 Table).

Maternal and labor characteristics and maternal and neonatal outcomes in respondents according to the ACOG classification were developed in the Table 1. Concerning the female and male sexual function, 96.3% (873/907) of the couple (man-woman) reported sexual activity at 6 months postpartum. The entire cohort consisted of heterosexual couples. The mean PISQ-12 score among the 873 women who reported sexual activity at 6 months was 38.6±7.1 and did not differ between midpelvic and low pelvic aOVD (p = 0.82) (Table 1). The mean IIEF-15 score was 63.3±11.1 and was not significantly different according to the fetal head station. Perineal pain and dyspareunia did not differ between midpelvic and low pelvic aOVD (p = 0.14 and p = 0.79, respectively) (Table 1).

**Table 1:** Characteristics of mothers and their labor and maternal and neonatal outcomes in respondents, according to the ACOG classification.

	Mid (N = 167)	Low (N = 740)	*P* value
**Maternal and labor characteristics**			
Maternal age, (years)^1^	29.2 ± 5.3	29.0 ± 4.7	0.59*
Geographic origin			0.34**
Europe, n (%)	156 (93.4)	699 (94.5)	
Sub-Saharan Africa, n (%)	5 (3.0)	8 (1.1)	
North Africa, n (%)	1 (0.6)	6 (0.8)	
Asia, n (%)	2 (1.2)	17 (2.3)	
Other, n (%)	3 (1.8)	10 (1.3)	
Married or living with a partner, n (%)	158 (95.8)	709 (96.2)	0.79**
Nulliparity, n (%)	118 (70.7)	564 (76.2)	0.13**
Previous cesarean delivery, n (%)	20 (42.6)	66 (37.5)	0.52**
Previous 3^rd^ or 4^th^-degree perineal lacerations, n (%)	0	1 (0.6)	0.60***
Previous depression, n (%)	5 (3.0)	38 (5.1)	0.25**
BMI before pregnancy (kg/m^22^)^1^	22.8 ± 4.1	22.7 ± 3.9	0.72*
Gestational weight gain (kg) ^1^	13.8 ± 4.5	13.3 ± 4.4	0.23*
Antenatal suspicion of macrosomia ^2^, n (%)	18 (10.8)	48 (6.5)	0.05**
Gestational age at delivery (weeks) ^1^	39.5 ± 1.5	39.4 ± 1.4	0.63*
Induced labor, n (%)	30 (18.0)	125 (16.9)	0.74**
Length of labor (min) ^1^	395.7 ± 179.1	388.4 ± 165.0	0.61*
Length of 2^nd^ stage (min) ^1^	103.3 ± 73.8	108.8 ± 67.3	0.35*
Active phase of 2^nd^ stage > 30 min, n (%)	51 (30.5)	267 (36.1)	0.28**
Dose of oxytocin (mUI) ^1^	1976.3 ± 2228.8	1620.9 ± 2084.8	0.05*
Epidural analgesia, n (%)	164 (98.2)	698 (94.5)	0.04**
Manual rotation, n (%)	30 (18.0)	81 (11.0)	0.01**
Persistent occiput			0.05**
Anterior, n (%)	139 (83.2)	662 (89.8)	
Posterior, n (%)	21 (12.6)	56 (7.6)	
Transverse, n (%)	7 (4.2)	19 (2.6)	
Indications for OVD			0.02**
Non reassuring FHR only, n (%)	86 (51.5)	301 (40.7)	
Arrested progress only, n (%)	51 (30.5)	313 (42.3)	
Non reassuring FHR and arrested progress, n (%)	30 (18.0)	129 (17.4)	
OVD in operating room, n (%)	12 (7.2)	4 (0.5)	<0.001***
Provider attending delivery			<0.001**
Senior obstetrician, n (%)	88 (54.7)	159 (21.7)	
Resident, n (%)	73 (45.3)	573 (78.3)	
Instrument type			
Vacuum, n (%)	13 (7.8)	237 (32.0)	<0.001**
Forceps, n (%)	20 (12.0)	38 (5.1)	<0.001***
Spatula, n (%)	140 (84.3)	485 (65.5)	<0.001**
Sequential use of instrument, n (%)	6 (3.6)	21 (2.8)	0.59***
**Maternal outcome**			
Cesarean section after failed OVD, n (%)	12 (7.2)	4 (0.5)	<0.001***
Episiotomy, n (%)	144 (87.3)	652 (88.1)	0.77**
3^rd^ or 4^th^-degree perineal lacerations, n (%)	3 (1.8)	25 (3.4)	0.30***
Perineal hematomas, n (%)	0	1 (0.1)	0.63***
Abscesses/hematoma required surgery, n (%)	1 (0.7)	3 (0.4)	0.69***
Postpartum hemorrhage (PPH), n (%)	35 (20.9)	128 (17.3)	0.27**
Severe PPH (blood loss>1500mL), n (%)	5 (3.0)	16 (2.2)	0.52***
Second-line therapies^3^, n (%)	1 (1.0)	1 (0.2)	0.25***
Blood transfusion, n (%)	7 (4.2)	12 (1.6)	0.04***
Infections^4^, n (%)	1 (0.7)	1 (0.1)	0.25***
Thromboembolic events, n (%)	0	2 (0.3)	0.50***
Maternal hospitalization in intensive care unit, n (%)	0	0	-
Severe maternal morbidity^5^, n (%)	13 (7.8)	64 (8.7)	0.71**
**Neonatal outcome**			
Birth weight≥4000 g, n (%)	12 (7.2)	38 (5.1)	0.30**
5-min Apgar score<7, n (%)	1 (0.6)	5 (0.7)	0.91***
pH<7.00, n (%)	4 (2.5)	10 (1.4)	0.32***
Transfer to NICU, n (%)	9 (5.4)	36 (4.9)	0.78**
NICU hospitalisation>24 h, n (%)	9 (5.4)	29 (3.9)	0.39**
Respiratory distress syndrome, n (%)	8 (4.8)	30 (4.1)	0.66**
Neonatal trauma^6^, n (%)	3 (1.8)	3 (0.4)	0.05***
Shoulder dystocia, n (%)	7 (4.4)	15 (2.0)	0.08***
Need for resuscitation or intubation, n (%)	0	8 (1.1)	0.18***
Severe neonatal morbidity^7^, n (%)	25 (15.0)	62 (8.4)	0.01**
**Registered variables at 6 months postpartum**			
Smoker (male), n (%)	47 (39.8)	218 (39.4)	0.92**
Chronic disease (male), n (%)	4 (3.4)	34 (6.1)	0.24**
Breastfeeding, n (%)	95 (93.1)	470 (93.3)	0.63**
Pelvic floor muscle training, n (%)	119 (77.8)	544 (79.1)	0.72**
Child in the parents’ bedroom during night, n (%)	14 (11.9)	63 (11.4)	0.89**
Episiotomy complications^8^, n (%)	54 (38.9)	245 (37.7)	0.80**
Perineal pain, n (%)	26 (17.2)	87 (12.7)	0.14**
Sexually active, n (%)	158 (94.6)	715 (96.6)	0.95**
Dyspareunia, n (%)	40 (23.9)	184 (24.8)	0.79**
EPDS^1^	6.5 ± 5.5	6.7 ± 6.1	0.72*
EPDS score ≥ 12	40 (23.9)	171 (23.1)	0.81**
PISQ-12 in sexually active women^1^	37.2 ± 7.8	37.1 ± 6.6	0.82*
IIEF-15 in sexually active men^1^	64.0 ± 10.3	63.2 ± 11.3	0.46*

^1^ Values are given as mean ± standard deviation.

^2^ Antenatal suspicion of macrosomia: fundal height measurement at delivery > 37cm and/or ultrasonographic fetal abdominal circumference > 90^th^ p. for gestational age and sex on Hadlock curves [22].

^3^ Second-line therapies were uterine compression sutures, uterine artery embolization, and peripartum hysterectomy for management of massive primary postpartum hemorrhage after failure of uterine massage and uterotonic agents to stop bleeding [2].

^4^ Infections were defined by the existence of at least one of the following criteria: endometritis, episiotomy infection and wound infection needed surgery [2].

^5^ Severe maternal morbidity was defined by the existence of at least one of the following criteria: third or fourth-degree perineal lacerations, perineal hematomas, cervical laceration, extension of uterine incision at cesarean section, PPH>1500 mL, surgical haemostatic procedure, uterine artery embolization, blood transfusion, infections (endometritis, episiotomy infection, wound infection needed surgery), thromboembolic events (deep vein thrombophlebitis and pulmonary embolism), hospitalization in intensive care unit, and maternal death [2].

^6^ Neonatal trauma was defined by the existence of at least one of the following criteria: fracture of the clavicle or a long bone, brachial plexus injury, and cephalhematoma [2].

^7^ Severe neonatal morbidity was defined by at least one of the following criteria: 5-minute Apgar score<7, umbilical artery pH < 7.00, need for resuscitation or intubation, neonatal trauma, intraventricular hemorrhage > grade 2, admission to the NICU (neonatal intensive care unit) for>24 hours, convulsions, sepsis, and neonatal death [2].

^8^ Episiotomy complications were defined by the existence of at least one of the following criteria: hematoma, abscess, scar disunion, or required surgery for episiotomy.

* Student t test,

** χ2 test,

*** Fisher exact test. Statistical significance was defined as a *P* value < 0.05.

EPDS: Edinburgh Postnatal Depression Scale [31]. PISQ-12: Pelvic Organ Prolapse Urinary Incontinence Sexual Questionnaire [28]. IIEF-15: International Index of Erectile Function [35].

In the multivariable linear regression analysis, attempted midpelvic delivery (compared with low pelvic) was not associated with female sexual dysfunction at 6 months (p = 0.89), controlling for maternal age, indications for aOVD, the American College classification, 3^rd^ or 4^th^-degree perineal lacerations, episiotomy complications, perineal pain, dyspareunia, and child in the parents' bedroom during night (Table 2). The PISQ-12 score was reduced on average of -0.10 points compared to low attempted delivery (95% confidence interval (CI) CI -1.57 to 1.38) (Table 2). Attempted midpelvic delivery (compared with low pelvic) was not also associated with male sexual dysfunction at 6 months (P = 0.76), controlling for maternal age, gestational age at delivery, the American College classification, indications for aOVD, instrument type, perineal pain, and smoker (male) (Table 3). The IIEF-15 score was reduced of -0.32 points compared to low attempted delivery (95% CI -2.43 to 1.79) (Table 3). Perineal pain, episiotomy complications and dyspareunia were each significantly associated with alterations of the female sexual function (Table 2). Forceps delivery and female perineal pain were significantly associated with alterations of the male sexual function (Table 3).

**Table 2:** Univariate and multiple linear regression analysis of female sexual function 6 months after midpelvic and low attempted operative vaginal delivery.

Variables ^1^	Female sexual function (PISQ-12 score)					
	Crude R (95% CI)	Effect size	*P* value	Adjusted R (95% CI)	Effect size	*P* value
ACOG classification						
Mid	Reference		-	Reference		-
Low	-0.14 (-1.36–1.08)	-0.22	0.82	-0.10 (-1.57–1.38)	-0.13	0.89
Indications for aOVD						
Arrested progress only	Reference		-	Reference		-
Non-reassuring FHR only	1.43 (0.40–2.46)	2.72	0.006	1.66 (0.45–2.87)	2.69	0.007
Non-reassuring FHR and arrested progress	0.45 (-0.90–1.81)	0.65	0.51	0.80 (-0.74–2.34)	1.02	0.31
3^rd^ or 4^th^-degree perineal lacerations	-2.54 (-5.26–0.18)	-1.83	0.06	-2.84 (-6.00–0.31)	-1.76	0.08
Episiotomy complications	-1.36 (-2.37- -0.35)	-2.64	0.008	-1.30 (-2.42- -0.17)	-2.28	0.02
Perineal pain	-3.43 (-4.79- -2.08)	-4.94	<0.001	-2.39 (-4.08- -0.70)	-2.77	0.006
Dyspareunia	-2.06 (-1.02- -3.11)	-1.92	<0.001	-1.51 (-2.81- -0.21)	-2.28	0.02
Child in the parents’ bedroom during night	-1.64 (-3.22- -0.06)	-2.03	0.04	1.50 (-3.25–0.26)	-1.68	0.09
Cste				36.6 (33.9–39.3)		

Values are crude and adjusted coefficient's linear regression (R) with 95% confidence intervals (CI).

^1^ Controlling for maternal age, ACOG classification, indications for aOVD, 3^rd^ or 4^th^-degree perineal lacerations, episiotomy complications, perineal pain, dyspareunia, and child in the parents' bedroom during night.

**Table 3:** Univariate and multiple linear regression analysis of male sexual function 6 months after midpelvic and low attempted operative vaginal delivery.

Variables ^1^	Male sexual function (IIEF-15 score)					
	Crude R (95% CI)	Effect size	*P* value	Adjusted R (95% CI)	Effect size	*P* value
Gestational age at delivery (per week)	-0.53 (-1.17–0.11)	-1.62	0.10	-0.60 (-1.20- -0.003)	-1.96	0.049
ACOG classification						
Mid	Reference		-	Reference		-
Low	-0.82 (-3.02–1.37)	-0.73	0.46	-0.32 (-2.43–1.79)	-0.30	0.76
Instrument type						
Other instrument	Reference		-	Reference		-
Forceps	-4.51 (-8.02- -1.01)	-2.52	0.01	-4.96 (-8.30- -1.62)	-2.91	0.004
Indications for aOVD						
Arrested progress only	Reference		-	Reference		-
Non-reassuring FHR only	3.52 (1.68–5.36)	3.75	<0.001	3.05 (1.29–4.81)	3.40	0.001
Non-reassuring FHR and arrested progress	1.76 (-0.63–4.16)	1.44	0.15	1.19 (-1.08–3.47)	1.03	0.30
Perineal pain	-2.45 (-4.98–0.08)	-1.90	0.06	-2.74 (-5.10- -0.38)	-2.28	0.02
Smoker (male)	1.30 (-0.32–2.92)	1.57	0.11	1.34 (-0.29–2.97)	1.61	0.11
Cste				82.5 (58.4–106.6)		

Values are crude and adjusted coefficient's linear regression (R) with 95% confidence intervals (CI).

^1^ Controlling for maternal age, gestational age at delivery, ACOG classification, indications for aOVD, instrument type, perineal pain, and smoker (male).

Concerning the assessment of maternal depressive symptoms at 6 months postpartum, the mean EPDS score was not significantly different between the groups (p = 0.72) (Table 1). Previous maternal PPD, BMI ≥ 30 kg/m^22^ before pregnancy, perineal pain, PISQ-12 and IIEF-15 scores were significantly different depending on the PPD status (Table 4). In multivariate analysis, attempted midpelvic (compared with low pelvic) delivery was not significantly associated with maternal symptoms of PPD (adjusted odds ratio (aOR) 0.95, 95% CI 0.58–1.53). BMI ≥ 30 kg/m^22^ before pregnancy and perineal pain were each significantly associated with maternal symptoms of PPD (Table 5). Correlation between IIEF-15 and PISQ-12 scores was 0.45 (95% CI 0.39 to 0.51), and correlation between EPDS and PISQ-12 scores was -0.31 (95% CI -0.37 to -0.24). Finally, small Cohen's d effect sizes were noted in all comparisons (female sexual function, maternal symptoms of PPD, male sexual function) concerning comparisons of midpelvic to low pelvic aOVD.

**Table 4:** Symptoms of postpartum depression according to characteristics of mothers and labor and maternal, paternal, and neonatal outcomes in women 6 months after midpelvic or low attempted operative vaginal delivery.

	Postpartum Depression (N = 211)	No-postpartum Depression (N = 603)	*P* value
**Maternal and labor characteristics**			
Maternal age^1^, (years)	29.2 ± 5.3	29.0 ± 4.6	0.61*
Multiparity, n (%)	49 (23.2)	155 (25.7)	0.47**
BMI ≥ 30 kg/m^2^ before pregnancy, n (%)	23 (11.0)	25 (4.2)	<0.001**
Gestational weight gain >20 kg, n (%)	19 (9.3)	47 (8.1)	0.58**
Previous postpartum depression, n (%)	23 (10.9)	18 (3.0)	<0.001**
Gestational age at delivery^1^ (weeks)	39.4 ± 1.5	39.4 ± 1.4	0.99*
Length of labor^1^ (min)	396.4 ± 169.0	390.2 ± 165.4	0.64*
2^nd^ stage>3 hours, n (%)	37 (17.5)	101 (16.8)	0.80**
Active phase of 2^nd^ stage > 30 min, n (%)	77 (36.5)	212 (35.2)	0.73**
Epidural analgesia, n (%)	201 (95.3)	576 (95.7)	0.80**
Persistent occiput position, n (%)			0.15**
Anterior	183 (87.1)	538 (89.5)	
Posterior	17 (8.1)	50 (8.3)	
Transverse	10 (4.8)	13 (2.2)	
ACOG classification, n (%)			0.37**
Mid	43 (20.4)	106 (17.6)	
Low	168 (79.6)	497 (82.4)	
Obstetrician performing delivery, n (%)			0.32**
Senior obstetrician	51 (24.8)	169 (28.3)	
Obstetric registrar	155 (75.2)	428 (71.7)	
Instrument type, n (%)			
Vacuum	60 (28.4)	161 (26.7)	0.63**
Forceps	18 (8.5)	34 (5.6)	0.14**
Spatula	140 (66.4)	421 (69.8)	0.35**
Sequential use of two instruments	6 (2.8)	14 (2.3)	0.67***
Indications for aOVD, n (%)			0.47**
Non-reassuring FHR only	88 (41.7)	261 (43.3)	
Arrested progress only	84 (39.8)	248 (41.0)	
Non-reassuring FHR and arrested progress	41 (19.4)	95 (15.7)	
**Maternal outcome**			
Cesarean delivery after failed operative vaginal delivery, n (%)	4 (1.9)	12 (2.0)	0.93***
Episiotomy, n (%)	181 (85.8)	532 (88.4)	0.32**
3^rd^ or 4^th^-degree perineal lacerations, n (%)	6 (2.8)	20 (3.3)	0.73**
PPH (blood loss>500mL), n (%)	34 (16.1)	114 (18.9)	0.37**
Severe PPH (blood loss>1500 mL), n (%)	6 (2.8)	14 (2.3)	0.67***
Abscesses/hematoma requiring surgery, n (%)	1 (0.5)	2 (0.3)	0.77***
Severe maternal morbidity, n (%)	17 (8.1)	53 (8.8)	0.74**
**Neonatal outcome**			
Birth weight > 4000 g, n (%)	15 (7.4)	32 (5.3)	0.33**
Neonatal trauma, n (%)	2 (1.0)	3 (0.5)	0.47***
Transfer to NICU, n (%)	9 (4.3)	33 (5.5)	0.49**
Severe neonatal morbidity, n (%)	18 (8.5)	62 (10.3)	0.46**
**Registered variables at 6 months postpartum**			
Tobacco (male), n (%)	71 (43.8)	180 (37.3)	0.14**
Chronic disease (male), n (%)	11 (6.8)	27 (5.6)	0.57**
Breastfeeding, n (%)	129 (90.8)	377 (94.0)	0.20**
Pelvic floor muscle training, n (%)	157 (77.3)	481 (80.0)	0.41**
Child in the parents’ bedroom during night, n (%)	19 (11.9)	54 (11.2)	0.82**
Episiotomy complications, n (%)	119 (62.0)	349 (61.9)	0.98**
Perineal pain, n (%)	36 (17.8)	73 (12.2)	0.04**
Dyspareunia, n (%)	69 (37.3)	177 (31.8)	0.31**
PISQ-12 in sexually active women^1^	34.5 ± 7.9	38.1 ± 6.0	<0.001*
IIEF-15 in sexually active men^1^	60.3 ± 14.3	64.5 ± 9.2	<0.001*

^1^ Values are given as mean ± standard deviation.

* Student t test,

** χ2 test,

*** Fisher exact test. Statistical significance was defined as a *P* value < 0.05.

EPDS: Edinburgh Postnatal Depression Scale [31]. PISQ-12: Pelvic Organ Prolapse Urinary Incontinence Sexual Questionnaire [28]. IIEF-15: International Index of Erectile Function [35].

**Table 5:** Univariate and multivariate logistic regression analysis of symptoms of postpartum depression 6 months after midpelvic or low attempted operative vaginal delivery.

	Maternal postpartum depression (N = 211)					
Variables^1^	Crude OR (95% CI)	Effect size	*P* value	Adjusted OR (95% CI)	Effect size	*P* value
BMI ≥ 30 kg/m^2^ before pregnancy	3.07 (1.66–5.71)	3.58	<0.001	2.86 (1.51–5.40)	3.22	0.001
Persistent occiput						
Anterior	Reference		-	Reference		-
Posterior	1.41 (0.77–2.59)	1.11	0.27	1.40 (0.75–2.62)	1.06	0.30
Transverse	3.08 (1.31–7.29)	2.58	0.01	2.89 (1.19–7.03)	2.34	0.02
ACOG classification						
Mid	Reference		-	Reference		-
Low	0.93 (0.59–1.47)	-0.31	0.75	0.95 (0.58–1.53)	-0.20	0.83
Indications for aOVD						
Arrested progress only	Reference		-	Reference		-
Non-reassuring FHR only	0.68 (0.45–1.01)	-1.83	0.06	0.63 (0.41–0.97)	-2.11	0.04
Non-reassuring FHR and arrested progress	1.12 (0.68–1.84)	0.45	0.65	1.08 (0.65–1.81)	0.30	0.76
Perineal pain	1.83 (1.13–2.95)	2.46	0.01	1.79 (1.10–2.91)	2.34	0.02

Values are crude and adjusted coefficient's logistic regression (OR) with 95% confidence intervals (CI).

^1^ Controlling for maternal age, body mass index (BMI) before pregnancy, previous postpartum depression, persistent occiput orientation of fetal head, ACOG classification, indications for aOVD, and perineal pain.

## Discussion

### Main findings

This study reports a prospective population-based cohort analysis of women 6 months after midpelvic or low pelvic aOVD, and male and female sexual dysfunction and maternal symptoms of PPD according to the fetal head station. We found that midpelvic aOVD was not significantly associated with a higher rate in male and female sexual dysfunction and maternal symptoms of PPD than attempted low pelvic aOVD at 6 months postpartum. After multivariable analysis, postpartum perineal pain was an independent risk factor for male or female sexual dysfunction and maternal symptoms of PPD at 6 months after aOVD.

### Interpretation

It is difficult to compare our results with the literature because previous studies of sexual function and maternal symptoms of PPD at 6 months or 1 year postpartum after OVD never detailed results according to the fetal head station at instrument's application [15,40]. The sample size of this prospective cohort (N = 907) was larger in size to other prospective [40–42] and retrospective studies [15,43] of maternal consequences after OVD. Our results are consistent with other findings in the literature concerning health problems after OVD, i.e. female sexual activity at 6 months postpartum [40,43–45], altered sexual function in women with postpartum perineal pain [15,42] or with postpartum dyspareunia [40,43,46], increased maternal postpartum depressive symptoms in women with persistent perineal pain [19,44,47,48]. Our data also included what the women and the men were saying concerning sexual function in postpartum period; therefore, this important factor in the assessment of the quality of life in couple was assessed. We showed that persistent female perineal pain 6 months after aOVD were significantly associated with alterations of the male sexual function. To our knowledge, no study has specifically studied male sexual function in postpartum period according to the fetal head station at aOVD.

### Strengths and limitations

The principal strength of this study is the use of validated instruments for male and female sexual dysfunction and maternal symptoms of PPD at 6 months postpartum in a large, prospective population-based cohort study with carefully characterized obstetric patients. This allowed a complete characterization of symptoms in this population. In particular, this is, to our knowledge, the first prospective population-based cohort study, that directly compares midpelvic and low aOVDs for male and female sexual dysfunction and maternal symptoms of PPD at 6 months postpartum. Obstetric situation with a potentially difficult OVD or perform a caesarean at full dilatation seems to present the perfect setting for a randomised trial, which probably failed to achieve due to recruitment that is not feasible in the antenatal period. Furthermore, when the fetus is at low pelvic station, OVD is not discussed for maternal or fetal indication, and must be preferred to caesarean delivery. As our results showed no difference between low pelvic and midpelvic OVD regarding inherent immediate maternal outcomes [2] and 6 months after OVD [3], the continued use of midpelvic delivery in appropriately selected candidates should be considered.

Our study has some limitations. First, determination of the station of the fetal head and thus classification of the OVDs is quite subjective and is influenced by fetal head position, molding, and time of assessment (before or after regional analgesia) [2]. Nevertheless, the prevalence of midpelvic aOVD was similar to that in other study [41], and the rates of induced labour, persistent occiput posterior or transverse, manual rotation, forceps and spatula, aOVD performed by senior obstetricians and aOVD in an operating room were significantly higher in the midpelvic compared to the lowpelvic aOVD group, as previously shown [2]. This suggests that the risk of contamination between the two groups was low [2]. Second, episiotomy and perineal tears of third/fourth degree were not found to be associated with alteration of female sexual function or dyspareunia at 6 months, consistent with some studies [41,49,50], but not with others [15,51]. These discrepancies may be explained by methodological differences. Several hypotheses may explain the discrepancies between these data concerning female sexual function after childbirth: memory bias for obstetric variables in retrospective study [15,51], or length of time in the postpartum period chosen for evaluation, which may be an important bias due to anatomical change [41]. Third, we reported a relative low rate of respondents (46.7%), but this rate was consistent with others large postpartum evaluations using mailed questionnaire [40,43]. It is plausible that participants declining to respond to a questionnaire at 6 months were at higher risk of sexual dysfunction and/or depressive symptoms than participants included in the study. Nevertheless, the non-respondents differed mainly from the respondents in their rate of neonatal morbidity, a factor that was not related to either male and female sexual dysfunction and symptoms of PPD at 6 months in our study. Four, no power calculations were done before the analysis of the questionnaire at 6 months because the study was based on post hoc analysis from a prospective population-based cohort study without any pre-specified hypothesis concerning covariates which should be associated with the outcome (sexual function and maternal symptoms of depression) at 6 months. Nonetheless, we would like to underline that our sample size was sufficient to show an effect size of 0.24 (considered as a small effect size) with a power of 80% assessed according to the observed incidence of male and female sexual dysfunction and symptoms of PPD at 6 months. Five, the decision most often faced to the obstetrician is, arguably, the choice between midpelvic aOVD and emergency cesarean section at full dilatation, rather than mid- vs lowpelvic aOVD. From this perspective, the absence of any data on outcomes for women having an emergency cesarean section at full dilatation is a limitation of the study.

## Conclusion

We found that both female and male sexual function as well as maternal depressive symptoms did not differ 6 months after childbirth among women who had experienced midpelvic and low pelvic aOVD. These findings at 6 months should help clinician to inform women and their partners about what to expect after a midpelvic aOVD. The data at 6 months postpartum are reassuring and need further studies at long-term to confirm these short-term data.

## Supporting information

S1 Questionnaire Questionnaire used at 6 months to assess female and male sexual function and symptoms of maternal postpartum depression. (DOC)

S1 Table Maternal and labor characteristics and maternal and neonatal outcomes for respondents and non-respondents. (DOC)
