# Supplementary material for: Sexual function and postpartum depression 6 months after attempted operative vaginal delivery according to fetal head station: A prospective population-based cohort study
Source: PLoS One. 2017 Jun 7;12(6):e0178915. doi: 10.1371/journal.pone.0178915 (PMC5462380; doi:10.1371/journal.pone.0178915)
Supplement: S1 Questionnaire — (DOC) [file pone.0178915.s001.doc]

# S1 Questionnaire.

**Pelvic Organ Prolapse/Urinary Incontinence Sexual Function Questionnaire (PISQ-12)**

Following are a list of questions about you and your partner’s sex life. All information is strictly confidential. Your confidential answers will be used only to help doctors understand what is important to patients about their sex lives. Please check the box that best answers the question for you. While answering the questions, consider your sexuality over the past six months.

*(from Rockwood G, et al. 2003)*

1. How frequently do you feel sexual desire? This feeling may include wanting to have sex, planning to have sex, feeling frustrated due to lack of sex…

□ Always □ Usually □ Sometimes □ Seldom □ Never

1. Do you climax (have an orgasm) when having sexual intercourse with your partner?

□ Always □ Usually □ Sometimes □ Seldom □ Never

1. Do you feel sexually excited (turned on) when having sexual activity with your partner?

□ Always □ Usually □ Sometimes □ Seldom □ Never

1. How satisfied are you with the variety of sexual activities in your current sex life?

□ Always □ Usually □ Sometimes □ Seldom □ Never

1. Do you feel pain during intercourse?

□ Always □ Usually □ Sometimes □ Seldom □ Never

1. Are you incontinent of urine (leak urine) with sexual activity?

□ Always □ Usually □ Sometimes □ Seldom □ Never

1. Does fear of incontinence (either stool or urine) restrict your sexual activity?

□ Always □ Usually □ Sometimes □ Seldom □ Never

1. Do you avoid sexual intercourse because of bulging in the vagina (either the bladder, rectum or vagina falling out)?

□ Always □ Usually □ Sometimes □ Seldom □ Never

1. When you have sex with your partner, do you have negative emotional reactions such as fear, disgust, shame or guilt?

□ Always □ Usually □ Sometimes □ Seldom □ Never

1. Does your partner have a problem with erections that affects your sexual activity?

□ Always □ Usually □ Sometimes □ Seldom □ Never

1. Does your partner have a problem with premature ejaculation that affects your sexual activity?

□ Always □ Usually □ Sometimes □ Seldom □ Never

1. Compared to orgasms you have had in the past, how intense are the orgasms you have had in the past six months?

□ Much less intense □ Less intense □ Same intensity

□ More intense □ Much more intense

**Edinburgh Postnatal Depression Scale (EPDS)**

As you have recently had a baby, we would like to know how you are feeling. Please UNDERLINE which comes closest to how you have felt IN THE PAST 7 DAYS, not just how you feel today.

1. I have been able to laugh and see the funny side of things as much as I always could.

0 – As much as I always could

1 – Not quite so much now.

2 – Definitely not so much now

3 – Not at all

1. I have looked forward with enjoyment to things.

0 – As much as I ever did

1 – Rather less than I used to

2 – Definitely less than I used to

3– Hardly at all

1. I have blamed myself unnecessarily when things went wrong.

3 – Yes, most of the time.

2 – Yes, some of the time

1 – Not very often

0 – No, never

1. I have been anxious or worried for no good reasons.

0 – No, not at all.

1. - Hardly, ever
2. – Yes, sometimes
3. - Yes, very often
4. I have felt scared or panicky for no very good reason.

3– Yes, quite a lot

2 – Yes, sometimes

1 – No, not much

0 – No, not at all

1. Things have been getting on top of me.

3– Yes, most of the time I haven’t been able to cope at all

2 - Yes, sometimes I haven’t been coping as well as usual

1 – No, most of the time I have coped quite will

0 – No, I have been coping as well as ever

1. I have been so unhappy that I have had difficulty sleeping

3– Yes, most of the time

2 – Yes, sometimes

1 – Not very often

0 – No, not at all

1. I have felt sad or miserable

3-Yes, most of the time

2- Yes, quite often

1. Not very often
2. No, not at all
3. I have been so unhappy that I have been crying

3-Yes, most of the time

1. 2- Yes, quite often

1 -Only occasionally

0 – No, not at all

1. The thought of harming myself has occurred to me.

3-Yes, quite often

2-Sometimes

1-Hardly ever

0-Never

*(from Cox JL. 1987 & 2003)*

**PERINEAL CONSIDERATIONS**

Did you perform postpartum pelvic floor exercises? ‭ YES ‭ NO

Do you have chronic perineal pain (perineum designates the skin and muscle around the vaginal and anal outlets)? ‭ YES ‭ NO

Did you have an episiotomy? ‭ YES ‭ NO

If yes, please answer each question, thinking about the complications concerning your episiotomy you have experienced:

Hematoma ‭ YES ‭ NO

Abscess ‭ YES ‭ NO

Scar disunion ‭ YES ‭ NO

Surgery ‭ YES ‭ NO

# BREASTFEEDING

Did you continue breastfeeding? ‭ YES ‭ NO

**International Index of Erectile Function Questionnaire (IIEF-15)**

These questions ask about the effects your erection problems have had on your sex life, over the past 4 weeks. Please answer the following questions as honestly and clearly as possible.

*(from Rosen RC, et al. 1997)*

**Mark ONLY one circle per question:**

1. Over the past 4 weeks, how often were you able to get an erection during sexual activity?

0 No sexual activity  0 Almost always or always 
0 Most times (much more than half the time)  0 Sometimes (about half the time) 
0 A few times (much less than half the time)  0 Almost never or never

2. Over the past 4 weeks, when you had erections with sexual stimulation, how often were your erections hard enough for penetration?

0 No sexual stimulation  0 Almost always or always 
0 Most times (much more than half the time)  0 Sometimes (about half the time) 
0 A few times (much less than half the time)  0 Almost never or never

Questions 3, 4 and 5 will ask about erections you may have had during sexual intercourse.

3. Over the past 4 weeks, when you attempted sexual intercourse, how often were you able to penetrate (enter) your partner?

0 Did not attempt intercourse  0 Almost always or always 
0 Most times (much more than half the time)  0 Sometimes (about half the time) 
0 A few times (much less than half the time)  0 Almost never or never

4. Over the past 4 weeks, during sexual intercourse, how often were you able to maintain your erection after you had penetrated (entered) your partner?

0 Did not attempt intercourse 0 Almost always or always
0 Most times (much more than half the time) 0 Sometimes (about half the time)
0 A few times (much less than half the time) 0 Almost never or never

5. Over the past 4 weeks, during sexual intercourse, how difficult was it to maintain your erection to completion of intercourse?

0 Did not attempt intercourse  0 Almost always or always 
0 Most times (much more than half the time)  0 Sometimes (about half the time) 0 A few times (much less than half the time)  0 Almost never or never

6. Over the past 4 weeks, how many times have you attempted sexual intercourse?

0 No attempts  0 1-2 attempts 
0 3-4 attempts  0 5-6 attempts 
0 7-10 attempts  0 11 or more attempts

7. Over the past 4 weeks, when you attempted sexual intercourse how often was it satisfactory for you?

0 Did not attempt intercourse  0 Almost always or always 
0 Most times (much more than half the time)  0 Sometimes (about half the time) 
0 A few times (much less than half the time)  0 Almost never or never

8. Over the past 4 weeks, how much have you enjoyed sexual intercourse?

0 No intercourse  0 Very highly enjoyable 
0 Highly enjoyable  0 Fairly enjoyable 
0 Not very enjoyable  0 Not enjoyable

9. Over the past 4 weeks, when you had sexual stimulation or intercourse how often did you ejaculate?

0 Did not attempt intercourse  0 Almost always or always 
0 Most times (more than half the time)  0 Sometimes (about half the time) 
0 A few times (much less than half the time)  0 Almost never or never

10. Over the past 4 weeks, when you had sexual stimulation or intercourse how often did you have the feeling of orgasm or climax (with or without ejaculation)?

0 No sexual stimulation or intercourse 0 Almost always or always
0 Most times (much more than half the time) 0 Sometimes (about half the time)
0 A few times (much less than half the time) 0 Almost never or never

Questions 11 and 12 ask about sexual desire. Let's define sexual desire as a feeling that may include wanting to have a sexual experience (for example, masturbation or intercourse), thinking about having sex or feeling frustrated due to a lack of sex.

11. Over the past 4 weeks, how often have you felt sexual desire?

0 Almost always or always  0 Most times (much more than half the time) 
0 Sometimes (about half the time)  0 A few times (much less than half the time) 
0 Almost never or never

12. Over the past 4 weeks, how would you rate your level of sexual desire?

0 Very high  0 High  0 Moderate  0 Low  0 Very low or none at all

13. Over the past 4 weeks, how satisfied have you been with you overall sex life?

0 Very satisfied  0 Moderately satisfied 
0 About equally satisfied and dissatisfied  0 Moderately dissatisfied 
0 Very dissatisfied

14. Over the past 4 weeks, how satisfied have you been with your sexual relationship with your partner?

0 Very satisfied 0 Moderately satisfied
0 About equally satisfied and dissatisfied 0 Moderately dissatisfied
0 Very dissatisfied

15. Over the past 4 weeks, how do you rate your confidence that you can get and keep your **erection**?

0 Very high 0 High 0 Moderate 
0 Low  0 Very low
