# Supplementary material for: Sexual function and postpartum depression 6 months after attempted operative vaginal delivery according to fetal head station: A prospective population-based cohort study
Source: PLoS One. 2017 Jun 7;12(6):e0178915. doi: 10.1371/journal.pone.0178915 (PMC5462380; doi:10.1371/journal.pone.0178915)
Supplement: S1 Table — (DOC) [file pone.0178915.s002.doc]

**Sexual function and postpartum depression 6 months after attempted operative vaginal delivery according to fetal head station: A prospective population-based cohort study.**

**Guillaume Ducarme^1,*^, Jean-François Hamel^2^, Stéphanie Brun^3^, Hugo Madar^3^, Benjamin Merlot^3^, Loïc Sentilhes^3^**

^1^ Department of Obstetrics and Gynecology, Centre Hospitalier Departemental, La Roche sur Yon, France

^2^ Clinical Research Center, Angers University Hospital, Angers, France

^3^ Department of Obstetrics and Gynecology, Bordeaux University Hospital, Bordeaux, France

Corresponding author: Guillaume DUCARME, MD, PhD

Department of Obstetrics and Gynaecology

Centre Hospitalier Departemental

85000 La Roche sur Yon, France.

Tel.: +33 251446570; fax: +33 251446404

E-mail address: [g.ducarme@gmail.com](mailto:g.ducarme@gmail.com)

**Short title:** Sexuality and depression after operative vaginal delivery.

S1 Table. Maternal and labor characteristics and maternal and neonatal outcomes for respondents and non-respondents.

|  | **Non-Respondents**  **(N=1,034)** | **Respondents**  **(N=907)** | P value |
| --- | --- | --- | --- |
| **Maternal and labor characteristics** |  |  |  |
| Maternal age (years)^1^ | 27.6 ± 5.2 | 29.0 ± 4.8 | <0.001* |
| Geographic origin |  |  | <0.001** |
| Europe, n (%) | 847 (85.2) | 855 (94.3) |  |
| Sub-Saharan Africa, n (%) | 30 (3.0) | 13 (1.4) |  |
| North Africa, n (%) | 12 (1.2) | 7 (0.8) |  |
| Asia, n (%) | 80 (8.1) | 19 (2.1) |  |
| Other, n (%) | 25 (2.5) | 13 (1.4) |  |
| Married or living with a partner, n (%) | 911 (91.9) | 867 (96.1) | <0.001** |
| Nulliparity, n (%) | 721 (72.4) | 682 (75.2) | 0.17** |
| Previous cesarean delivery, n (%) | 116 (42.2) | 86 (38.6) | 0.41** |
| Previous 3^rd^ or 4^th^-degree perineal lacerations, n (%) | 2 (0.7) | 1 (0.4) | 0.69*** |
| Previous depression, n (%) | 42 (4.2) | 43 (4.8) | 0.58** |
| BMI before pregnancy (kg/m²)^1^ | 23.4 ± 13.6 | 22.7 ± 3.9 | 0.13* |
| Gestational weight gain (kg)^1^ | 13.8 ± 5.8 | 13.4 ± 4.4 | 0.13* |
| Antenatal suspicion of macrosomia^2^, n (%) | 87 (8.7) | 66 (7.3) | 0.25** |
| Gestational age at delivery (weeks)^1^ | 39.3 ± 1.6 | 39.4 ± 1.4 | 0.17* |
| Induced labor, n (%) | 198 (19.9) | 155 (17.1) | 0.12** |
| Length of labor (min)^1^ | 378.6 ± 173.4 | 389.7 ± 167.6 | 0.16* |
| Length of active phase of 2^nd^ stage (min)^1^ | 23.8 ± 13.0 | 24.9 ± 12.9 | 0.06* |
| Dose of oxytocin (mUI)^1^ | 1860.8 ± 2489.0 | 1686.3 ± 2117.3 | 0.10* |
| Epidural analgesia, n (%) | 928 (93.2) | 862 (95.1) | 0.07** |
| Manual rotation, n (%) | 110 (11.3) | 111 (12.3) | 0.48** |
| Persistent occiput |  |  | 0.33** |
| Anterior, n (%) | 856 (86.4) | 801 (88.6) |  |
| Posterior, n (%) | 103 (10.4) | 77 (8.5) |  |
| Transverse, n (%) | 32 (3.2) | 26 (2.9) |  |
| Indications for OVD |  |  | 0.06** |
| Non reassuring FHR only, n (%) | 436 (43.8) | 387 (42.7) |  |
| Arrested progress only, n (%) | 339 (34.0) | 361 (39.8) |  |
| Non reassuring FHR and arrested progress, n (%) | 218 (21.9) | 159 (17.5) |  |
| OVD in operating room, n (%) | 23 (2.3) | 16 (1.8) | 0.40*** |
| Provider attending delivery |  |  | 0.47** |
| Senior obstetrician, n (%) | 258 (26.1) | 247 (27.7) |  |
| Resident, n (%) | 727 (73.8) | 646 (72.3) |  |
| Instrument type |  |  |  |
| Vacuum, n (%) | 303 (30.5) | 250 (27.6) | 0.16** |
| Forceps, n (%) | 62 (6.2) | 58 (6.4) | 0.89** |
| Spatula, n (%) | 662 (66.7) | 625 (68.9) | 0.29** |
| Sequential use of instrument, n (%) | 46 (4.6) | 27 (3.0) | 0.06** |
| ACOG Classification |  |  | 0.05** |
| Mid, n (%) | 220 (22.1) | 167 (18.4) |  |
| Low, n (%) | 776 (77.9) | 740 (81.6) |  |
| **Maternal outcome** |  |  |  |
| Cesarean section after failed OVD, n (%) | 23 (2.3) | 16 (1.8) | 0.40*** |
| Episiotomy, n (%) | 850 (86.1) | 796 (88.0) | 0.23** |
| 3^rd^ or 4^th^-degree perineal lacerations, n (%) | 24 (2.4) | 28 (3.1) | 0.59*** |
| Perineal hematomas, n (%) | 1 (0.1) | 1 (0.1) | 0.95*** |
| Abscesses/hematoma required surgery, n (%) | 8 (0.8) | 4 (0.5) | 0.30*** |
| Postpartum haemorrhage (PPH), n (%) | 158 (15.9) | 163 (17.9) | 0.09** |
| Severe PPH (blood loss>1500mL), n (%) | 13 (1.3) | 21 (2.3) | 0.10*** |
| Second-line therapies^3^, n (%) | 4 (0.7) | 2 (0.2) | 0.30*** |
| Blood transfusion, n (%) | 17 (1.7) | 19 (2.1) | 0.53*** |
| Infections^4^, n (%) | 2 (0.2) | 0 | 0.30*** |
| Thromboembolic events, n (%) | 2 (0.2) | 2 (0.2) | 0.92*** |
| Maternal hospitalization in intensive care unit, n (%) | 1 (0.1) | 0 | 0.33*** |
| Severe maternal morbidity^5^, n (%) | 83 (8.3) | 77 (8.5) | 0.90** |
| **Neonatal outcome** |  |  |  |
| Birth weight≥4,000 g, n (%) | 50 (5.0) | 50 (5.5) | 0.63** |
| 5-min Apgar score<7, n (%) | 11 (1.1) | 6 (0.7) | 0.30*** |
| pH<7.00, n (%) | 16 (1.6) | 14 (1.6) | 0.90*** |
| Transfer to NICU, n (%) | 84 (8.4) | 45 (5.0) | 0.003** |
| NICU hospitalisation>24 h, n (%) | 74 (7.4) | 38 (4.2) | 0.003** |
| Respiratory distress syndrome, n (%) | 33 (3.3) | 38 (4.2) | 0.32** |
| Neonatal trauma^6^, n (%) | 7 (0.7) | 6 (0.7) | 0.91*** |
| Shoulder dystocia, n (%) | 30 (3.1) | 22 (2.5) | 0.41*** |
| Need for resuscitation or intubation, n (%) | 5 (0.5) | 8 (0.9) | 0.31*** |
| Seizures, n (%) | 1 (0.1) | 4 (0.4) | 0.15*** |
| Severe neonatal morbidity^7^, n (%) | 127 (12.8) | 87 (9.6) | 0.03** |

^1^ Values are given as mean ± standard deviation.

^2^ Antenatal suspicion of macrosomia: fundal height measurement at delivery > 37cm and/or ultrasonographic fetal abdominal circumference > 90^th^ p. for gestational age on Hadlock curves [22].

^3^ Second-line therapies were uterine compression sutures, uterine artery embolization, and peripartum hysterectomy for management of massive primary postpartum haemorrhage after failure of uterine massage and uterotonic agents to stop bleeding [2].

^4^ Infections were defined by the existence of at least one of the following criteria: endometritis, episiotomy infection and wound infection needed surgery [2].

^5^ Severe maternal morbidity was defined by the existence of at least one of the following criteria: third or fourth-degree perineal lacerations, perineal hematomas, cervical laceration, extension of uterine incision at caesarean section, PPH>1500 mL, surgical haemostatic procedure, uterine artery embolization, blood transfusion, infections (endometritis, episiotomy infection, wound infection needed surgery), thromboembolic events (deep vein thrombophlebitis and pulmonary embolism), hospitalization in intensive care unit, and maternal death [2].

^6^ Neonatal trauma was defined by the existence of at least one of the following criteria: fracture of the clavicle or a long bone, brachial plexus injury, and cephalhematoma [2].

^7^ Severe neonatal morbidity was defined by at least one of the following criteria: 5-minute Apgar score<7, umbilical artery pH < 7.00, need for resuscitation or intubation, neonatal trauma, intraventricular haemorrhage > grade 2, admission to the NICU (neonatal intensive care unit) for>24 hours, convulsions, sepsis, and neonatal death [2].

* Student t test, ** χ2 test, *** Fisher exact test. Statistical significance was defined as a *P* value < 0.05.
